# Assessment of Health-Related Quality of Life in Patients with Active Versus Inactive Adult-Onset Still’s Disease: Data from the PRO-AOSD Survey During the COVID-19 Pandemic

**DOI:** 10.3390/jcm14217848

**Published:** 2025-11-05

**Authors:** Norbert Blank, Ioana Andreica, Jürgen Rech, Zekayi Sözen, Eugen Feist

**Affiliations:** 1 Division of Rheumatology, Department of Internal Medicine V, University Hospital Heidelberg, 69120 Heidelberg, Germany; 2 Center for Rare Diseases Heidelberg (ZSEHD), University Hospital Heidelberg, 69120 Heidelberg, Germany; 3 Department of Rheumatology, Ruhr-Universität Bochum, 44780 Bochum, Germany; ioana.andreica@elisabethgruppe.de; 4 Rheumazentrum Ruhrgebiet, 44649 Herne, Germany; 5 Department of Internal Medicine 3—Rheumatology and Immunology, Friedrich-Alexander University (FAU) Erlangen-Nürnberg and Universitätsklinikum Erlangen, 91054 Erlangen, Germany; juergen.rech@uk-erlangen.de; 6 Deutsches Zentrum für Immuntherapie (DZI), Friedrich-Alexander-Universität Erlangen-Nürnberg and Uniklinikum Erlangen, 91054 Erlangen, Germany; 7 Center for Rare Diseases Erlangen (ZSEER), Friedrich-Alexander-Universität Erlangen-Nürnberg and Uniklinikum Erlangen, 91054 Erlangen, Germany; 8 Novartis Pharma AG, CH-4056 Basel, Switzerland; zek_soez@hotmail.com; 9 Department of Rheumatology and Clinical Immunology, Helios Fachklinik Vogelsang-Gommern Klinik für Rheumatologie, 39245 Gommern, Germany; eugen.feist@helios-gesundheit.de; 10 Experimental Rheumatology, Otto-von-Guericke Universität Magdeburg, 39106 Magdeburg, Germany

**Keywords:** mental health, qualitative research, rheumatology

## Abstract

**Background/Objectives**: To report patients’ perspectives on the impact of adult-onset Still’s disease (AOSD) on their health-related quality of life (HRQoL), work productivity and the effect of coronavirus (COVID)-19, using data from the PRO-AOSD (patient-reported outcomes adult-onset Still’s disease) survey in Germany. **Methods**: The PRO-AOSD survey comprised blinded patient and physician surveys. An additional post-hoc analysis was performed to determine the relationship between HRQoL and disease activity (defined per C-reactive protein and Physician’s Global Assessment data). The following outcomes were reported: patients’ perspectives on the impact of AOSD on their physical and mental HRQoL and work productivity, outcomes for patients with active versus inactive disease, and the effect of COVID-19 on their general health and work productivity. **Results**: Adult patients with AOSD were recruited from 19 centers in Germany. A total of 124 patients were included: 59.7% (74/124) were female, and the mean age of diagnosis was 38.2 years. Reported HRQoL was impaired in almost all domains, especially physical health. For the 58 patients whose data enabled categorization into active (31.0%, 18/58) versus inactive disease (69.0%, 40/58), patients with active disease reported significantly worse outcomes in the following (*p* < 0.001): likelihood of perceiving their health as excellent or similar to other people’s health, severity of pain in the past month, and the ability to complete strenuous activities. Although all patients were of working age, not all were employed (60.5%, 75/124). Many patients felt that compared to their peers, they were more burdened by the COVID-19 pandemic (33.9%) and were more afraid of contracting COVID-19 (49.2%). **Conclusions**: Patients with AOSD suffered from impaired HRQoL, which was worsened by active disease and the COVID-19 pandemic.

## 1. Introduction

Adult-onset Still’s disease (AOSD) is a rare multisystemic autoinflammatory disease (AID) of unknown etiology, which occurs in adults [1,2,3,4]. It is typically considered a diagnosis of exclusion and has a significant diagnostic delay [2,5]. AOSD is characterized by a triad of high-spiking fever, evanescent salmon-colored skin rash and arthritis, often accompanied by elevated acute-phase reactants [1,2,5]. Systemic juvenile idiopathic arthritis (sJIA), which is Still’s disease with childhood onset, is considered to be part of the same disease spectrum as AOSD [6].

AOSD follows a variable course including monocyclic (characterized by a single systemic episode completely resolving within months), polycyclic (associated with intermittent flares and characterized by complete remissions that can last up to a couple of years), and chronic (continuous symptoms and persistently active disease) patterns [3,5]. Treatment for AOSD includes corticosteroids (CSs), conventional synthetic disease modifying anti-rheumatic drugs (csDMARDs), and biological DMARDs (bDMARDs) [7].

Specific serum biomarkers can be used to assess inflammation in AOSD and high levels of C-reactive protein (CRP) are typically observed during disease flares and other inflammatory responses [7,8,9]. The Physician’s Global Assessment of disease activity (PGA) is a key outcome measure in Still’s disease and has been found to be a valid and reliable indicator of overall disease activity [10,11,12].

Health-related quality of life (HRQoL) measures assess the patient’s perception of the effect of disease and treatment on the physical, mental, and social aspects of their daily life [13]. The relevance of patient-reported outcomes (PROs) measuring HRQoL is being increasingly emphasized, as they provide multidimensional information on a patient’s disease, including insights into which health outcomes are most important to patients [14,15]. Despite the absence of a comprehensive evaluation of HRQoL in AOSD, it is well recognized that this disease imposes a significant burden on patients, both those with active disease and those in remission [16,17]. A recent study found that, compared with healthy individuals, patients with AOSD reported a significant reduction in most HRQoL PROs, particularly in the domains physical functioning and limitations due to physical health, and reported a perceived decline in their current health status by 25% compared with their ideal state [13]. In AOSD, PRO-based HRQoL assessment may be particularly useful given the disease’s clinical heterogeneity and the consequential diagnostic delay [5,17].

Given the underlying systemic inflammation and the need for immunosuppressive therapies, patients with AOSD may be particularly vulnerable to infections such as coronavirus (COVID)-19, further impacting the disease’s impact on overall health and quality of life [18,19,20]. The COVID-19 pandemic has greatly impacted patients with rheumatic diseases, and patients with AOSD are no exception [19]. Although several previous studies have assessed HRQoL and the effects of COVID-19 infection in rheumatic diseases [21,22,23], data from patients with AOSD are limited.

The PRO-AOSD (patient-reported outcomes adult-onset Still’s disease) survey is a blinded survey of symptoms and physical impairments, HRQoL, work productivity and the effect of COVID-19 on patients with AOSD in Germany. Data from this survey on patients’ perceptions of their symptoms and impairment, compared with the assessments of their treating physicians, have been reported [24].

Here, we report patients’ perspectives on the impact of AOSD on their physical and mental HRQoL and work productivity, as well as the impact of the COVID-19 pandemic on their general health and work productivity. We also explored possible correlations between HRQoL and disease activity.

## 2. Materials and Methods

### 2.1. Study Design and Patients

Patients were recruited from 19 centers in Germany to participate in this study, which included two PRO-AOSD surveys containing validated questionnaires supplemented with individual questions. One was completed by patients with AOSD using electronic tablets on the day of their appointment, and the other was completed online by physicians following the patient’s appointment. The HRQoL section (SF-36v2 questions) and COVID-19 section in the patient survey were designed with preselected response options, allowing patients to indicate their agreement by selecting the most applicable choice. SF-36 domains included in the HRQoL section were general state of health (general state of health perception), physical pain, physical health (physical functioning and physical role functioning), mental health and vitality (emotional role functioning, social role functioning, psychological well-being and vitality). The questions included in the COVID-19 section were developed specifically for this study. More detailed methods for the patient and physician survey have been described [24].

Outcomes reported here include patient demographics (age, weight, height, body mass index [BMI], current smoker, age at diagnosis, time to diagnosis), physical and mental HRQoL (SF-36v2 survey responses), impact of AOSD on HRQoL before COVID-19, effects of COVID-19 on the patient’s general health and work productivity, and informedness and information behavior.

An additional post-hoc analysis was performed to determine the relationship between HRQoL and disease activity, as assessed by CRP levels and PGA score. The distinction between patients with active and inactive disease was deemed essential to avoid masking the effects of disease activity within the overall group; we aimed to more accurately interpret the impact of disease activity on patients’ HRQoL by separately analyzing the data for these patients. The internally agreed definitions of inactive disease were CRP levels < 10 mg/L and PGA = 0, and active disease were CRP levels ≥ 10 mg/L and PGA > 0. PGA was scored on a 0–10 numerical scale.

### 2.2. Statistical Analysis

For some questions, the number of survey responses deviates from the total sample size due to missing or implausible data. In these cases, the deviating sample size is indicated.

The examination of group differences for smokers (female and male) was conducted using Chi-squared test and Fisher’s exact test. The examination of group differences for age, weight, height, BMI and age at diagnosis for males and females, as well as inactive disease and active disease, was conducted using independent *t*-tests and group differences. Due to unequal group sizes and partial violation of the assumptions of homogeneity of variances and normal distribution, the alternative Welch test was also calculated. The results of the Welch tests are reported here. The effect size is reported using Cohen’s *d*; small, moderate and large effects were defined as d = 0.2, 0.5 and 0.8, respectively. A *p*-value of  <0.05 was considered statistically significant. All analyses were conducted using JASP software (University of Amsterdam, The Netherlands), with the latest version available at the time of each analysis (versions: 0.17.1; 0.18.3; 0.19.1 and 0.19.2).

### 2.3. Patient and Public Involvement

Patients and/or the public were not involved in development of the research question, the design of this study, or the recruitment to and conduct of the study. Written informed consent was obtained from all patients before the study.

### 2.4. Ethics Approval

The study was approved by the ethics committee of the Otto von Guericke University, Medical Faculty and University Hospital, Magdeburg, Germany (approval code: 99/20, approval date: 21 July 2020); all participating institutions were covered by this ethics approval.

## 3. Results

### 3.1. Patient Demographics

The PRO-AOSD study included data from 124 adult patients with confirmed AOSD. The first patient completed the survey on 8 March 2021 and the last patient on 14 November 2022. Overall, 59.7% (74/124) of patients were female and 40.3% (50/124) of patients were male; the mean age of the entire study cohort was 45.5 years (SD 14.7; Table 1). The mean BMI of the study cohort was 27.0 kg/m^2^ (SD 5.8), and a total of 14.5% (18/124) of patients were smokers (Table 1).

#### 3.1.1. Time to Diagnosis

The mean age at onset of symptoms was 36 years and the mean age at diagnosis was 38.2 years (Table 1, *n* = 123). Patients experienced symptoms for a median duration of 0.25 years, prior to receiving a diagnosis of AOSD (*n* = 122). Most patients were aged between 30 to 49 years old at the onset of AOSD symptoms and diagnosis (Supplementary Figure S1).

#### 3.1.2. Disease Activity Determined by CRP Levels and PGA Scores

CRP and PGA score data were available for 58 of 124 patients; of these, 31.0% (18/58) were classified as having active disease and 69.0% (40/58) were classified as having inactive disease. From those classified as having active disease, 66.7% (12/18) were female and 33.3% (6/18) were male; 11.1% (2/18) were smokers (Table 1). Patient demographics were generally well balanced between patients with active and inactive disease (Table 1).

#### 3.1.3. Disease Course

**Figure 1:**
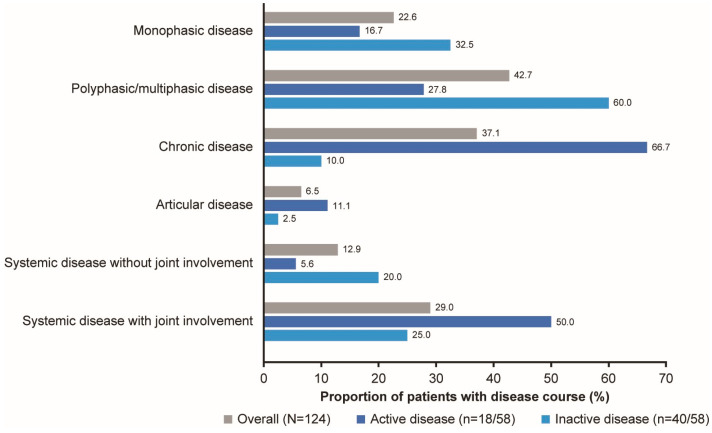
Physician-reported disease course of patients with AOSD. Active disease was defined as CRP levels ≥ 10 mg/L and PGA > 0, and inactive disease was defined as CRP levels < 10 mg/L and PGA = 0. AOSD, adult-onset Still’s disease; CRP, C-reactive protein; PGA, Physician’s Global Assessment of disease activity.

Overall, 42.7% (53/124) of patients were reported as having polyphasic/multiphasic disease, 37.1% (46/124) as having chronic disease and 22.6% (28/124) as having monophasic disease, with almost a third reported as having systemic disease with joint involvement (29.0%, 36/124) by physicians. Most patients with active disease (66.7%, 12/18) were reported as having chronic disease by physicians, whereas most patients with inactive disease (60.0%, 24/40) were reported as having a polyphasic/multiphasic disease course (Figure 1). Half of the patients with active disease (50.0%, 9/18) and one quarter of patients with inactive disease (25%, 10/40) were reported as having a systemic disease course with joint involvement by physicians (Figure 1).

### 3.2. HRQoL

#### 3.2.1. General State of Health

Overall, the mean score for the general state of health domain of the SF-36 questionnaire was 2.4 (1.2) (Supplementary Table S1). A total of 41.9% (52/124) of patients described their general state of health as ‘good’ and 34.7% (43/124) as ‘less good’, with only 2.4% (3/124) describing their general state of health as ‘excellent’ and 8.9% (11/124) as ‘bad’. Patients with active disease reported a general state of health that was significantly worse than those with inactive disease (*p* = 0.002; Cohen’s *d* effect size = 0.9). The most common responses were ‘good’ or ‘less good’ (44.4% each, 8/18) for patients with active disease, whereas half of the patients with inactive disease (50%, 20/40) described their general health status as ‘good’.

Around one-third of patients (33.9%, 42/124) reported that their current state of health was ‘about the same state as a year ago’. When describing their current state of health compared with the previous year, the difference observed between patients with active versus inactive disease was not statistically significant. The most common responses were ‘currently slightly worse than a year ago’ (33.3%, 6/18) for patients with active disease and ‘about the same as the previous year’ (42.5%, 17/40) for patients with inactive disease.

**Figure 2:**
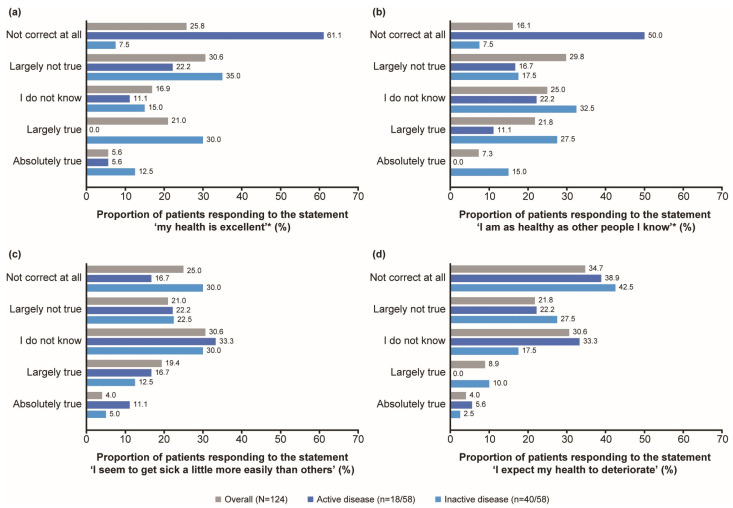
Patients’ general health status, as reported by patient-reported outcome measures, based on the statement (**a**) ‘my health is excellent’, (**b**) ‘I am as healthy as other people I know’, (**c**) ‘I seem to get sick a little more easily than others’ and (**d**) ‘I expect my health to deteriorate’. * *p* < 0.001 for active vs. inactive groups. Active disease was defined as CRP levels ≥ 10 mg/L and PGA > 0, and inactive disease was defined as CRP levels < 10 mg/L and PGA = 0. CRP, C-reactive protein; PGA, Physician’s Global Assessment of disease activity.

Most patients did not perceive their health to be excellent or themselves as healthy as other people they know. Around 30% of patients responded ‘largely not true’ to the statements ‘my health is excellent’ (30.6%, 38/124) and ‘I am as healthy as other people I know’ (29.8%, 37/124) (Figure 2a,b). Patients with active disease reported a health state that was significantly worse than those with inactive disease in terms of their level of agreement with the statement ‘my health is excellent’ (*p* < 0.001; Cohen’s *d* effect size = 1.2). The most common responses were ‘not correct at all’ (61.1%, 11/18) for patients with active disease and ‘largely not true’ (35.0%, 14/40) for patients with inactive disease (Figure 2a). Patients with active disease reported a health state that was significantly worse than those with inactive disease in terms of their level of agreement with the statement ‘I am as healthy as other people I know’ (*p* < 0.001; Cohen’s *d* effect size = 1.2). The most common responses were ‘not correct at all’ (50.0%, 9/18) for patients with active disease and ‘I do not know’ (32.5%, 13/40) or ‘largely true’ (27.5%, 11/40) for patients with inactive disease (Figure 2b).

Most patients did not believe that they get sick more easily, nor did they expect their health to deteriorate. One-quarter of patients (25.0%, 31/124) responded ‘not correct at all’ to the statement ‘I seem to get sick a little more easily than others’ and 34.7% (43/124) of patients responded ‘not correct at all’ to the statement ‘I expect my health to deteriorate’ (Figure 2c,d). When responding to the statements ‘I seem to get sick a little more easily than others’ and ‘I expect my health to deteriorate’, the differences observed between patients with active and inactive disease were not statistically significant.

#### 3.2.2. Pain

Overall, the mean score for the physical pain domain of the SF-36 questionnaire was 1.7 (1.3) (Supplementary Table S1). A total of 37.9% (47/124) of patients reported that they had experienced a moderate severity of pain in the past month. Patients with active disease reported pain that was significantly more severe than those with inactive disease (*p* < 0.001; Cohen’s *d* effect size = 1.2). The most common responses were ‘moderate pain’ (44.4%, 8/18) or ‘strong’ pain (22.2%, 4/18) for patients with active disease and ‘no pain’ (47.5%, 19/40) or ‘moderate pain’ (32.5%, 13/40) for those with inactive disease.

A total of 3.2% (4/124) of patients reported that pain has prevented them from completing daily activities at work and home in the past month. Patients with active disease, versus those with inactive disease, reported that their ability to complete daily activities was significantly more limited by pain (*p* < 0.001; Cohen’s *d* effect size = 1.3). For patients with active disease, the most common response was to report a ‘moderate’ limitation in their daily activities due to their pain in the past month (38.9%, 7/18). For those with inactive disease, half of patients responded that their activities were ‘not at all’ impacted by pain (50.0%, 20/40) and a quarter responded they experienced ‘somewhat’ of a limitation (25.0%, 10/40).

#### 3.2.3. Physical Health

**Figure 3:**
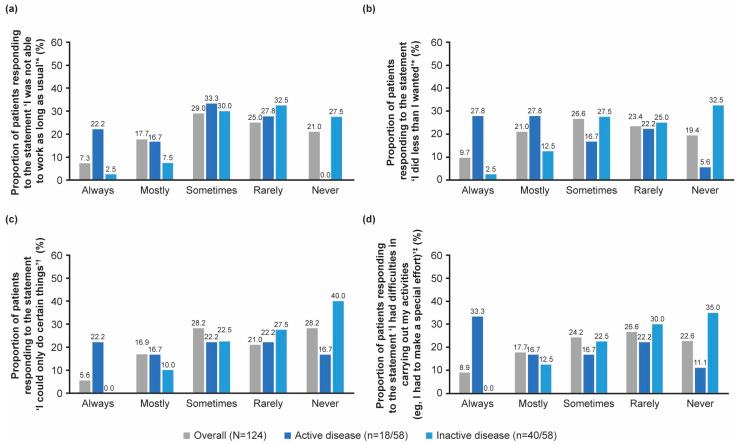
The impact of patients’ physical health status on their ability to complete work or everyday activities at work or home in the past month, as reported by patient-reported outcome measures, based on the statement (**a**) ‘I was not able to work as long as usual’, (**b**) ‘I did less than I wanted’, (**c**) ‘I could only do certain things’ and (**d**) ‘I had difficulties in carrying out my activities (e.g., I had to make a special effort)’. * *p* = 0.002 for active vs. inactive groups; ^†^ *p* = 0.011 for active vs. inactive groups; ^‡^ *p* = 0.003 for active vs. inactive groups. Active disease was defined as CRP levels ≥ 10 mg/L and PGA > 0, and inactive disease was defined as CRP levels < 10 mg/L and PGA = 0. CRP, C-reactive protein; PGA, Physician’s Global Assessment of disease activity.

Overall, the mean score for the physical health domain of the SF-36 questionnaire was 2.6 (1.4) (Supplementary Table S1). Only 21% (26/124) of patients reported that their physical health ‘never’ affects their ability to work for a usual amount of time, with 25.0% (31/124) of patients reporting that their ability was ‘rarely’ impacted and 7.3% (9/124) of patients reporting that their ability was ‘always’ impacted (Figure 3a). On average, patients with active disease reported that their ability to work for a usual amount of time in the past month was significantly more frequently impacted than those with inactive disease (*p* = 0.002; Cohen’s *d* effect size = −1.0). Although similar proportions of patients with active (33.3%, 6/18) versus inactive (30.0%, 12/40) disease responded that their ability to work a usual amount was ‘sometimes’ impacted by their physical health, 22.2% (4/18) of patients with active disease responded ‘always’, and 0% (0/18) of patients responded ‘never’, versus 2.5% (1/40) of patients with inactive disease who responded ‘always’ and 27.5% (11/40) who responded ‘never’ (Figure 3a).

Patients with active disease were significantly more affected than those with inactive disease when asked if they did less than they wanted (*p* = 0.002, Cohen’s *d* effect size = −1.0), were only able to do certain things (*p* = 0.011, Cohen’s *d* effect size = −0.8), or had difficulties in carrying out activities (in the past month) due to their physical health (*p* = 0.003, Cohen’s *d* effect size = −1.0) (Figure 3b–d).

For information on the impact of patients’ physical health status on their ability to complete general activities, such as bathing or dressing themselves, or lifting or carrying shopping bags, as reported by patient-reported outcome measures, refer to Supplementary Figure S2.

#### 3.2.4. Mental Health and Vitality

**Figure 4:**
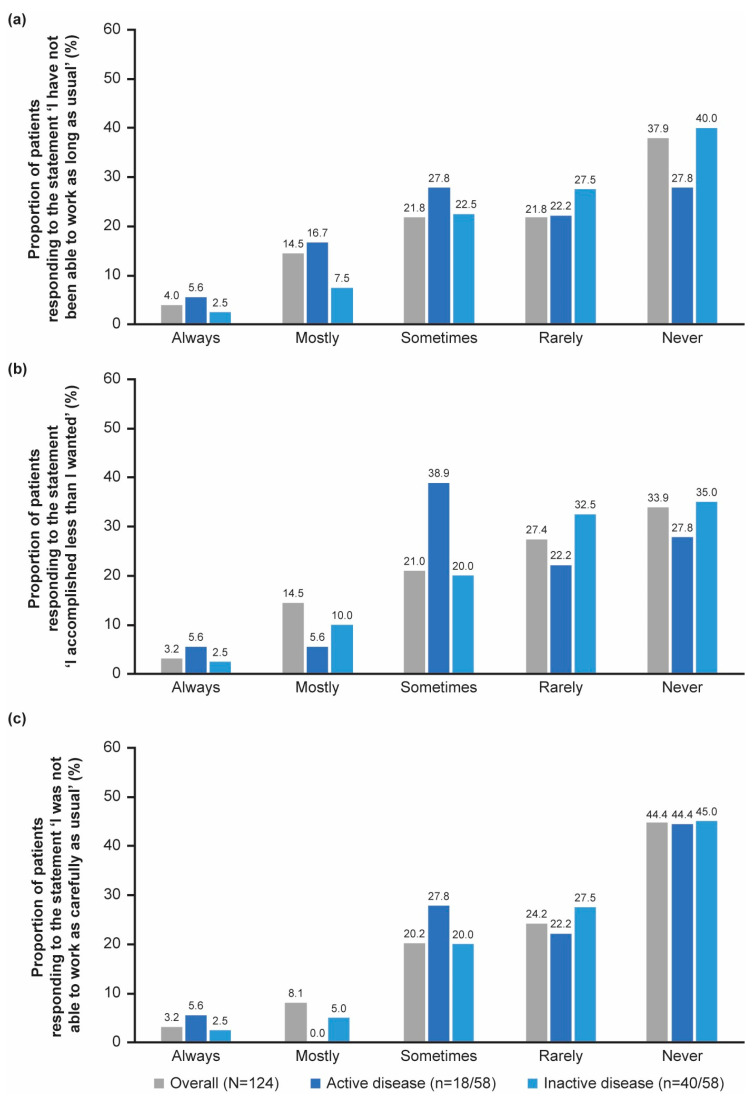
The impact of patients’ mental health status (e.g., feeling depressed or anxious) on their ability to complete work or everyday activities at work or home in the past month, as reported by patient-reported outcome measures, based on the statement (**a**) ‘I have not been able to work as long as usual’, (**b**) ‘I accomplished less than I wanted’ and (**c**) ‘I was not able to work as carefully as usual’. Active disease was defined as CRP levels ≥ 10 mg/L and PGA > 0, and inactive disease was defined as CRP levels < 10 mg/L and PGA = 0. CRP, C-reactive protein; PGA, Physician’s Global Assessment of disease activity.

Overall, the mean score for the mental health and vitality domain of the SF-36 questionnaire was 2.3 (1.2) (Supplementary Table S1). A total of 41.9% (52/124) of patients described themselves as mostly happy and 8.9% (11/124) said that they felt mostly discouraged and sad (Supplementary Figure S3a,b). Patients mainly responded that their mental health status did not impact their ability to work as long or carefully as usual, or to accomplish as much as they wanted (Figure 4a–c). A trend towards mental health affecting ability to work as long or as carefully as usual was observed in patients with active disease but not in those with inactive disease. However, these differences were not statistically significant.

Overall, 29.8% (37/124) of patients responded ‘mostly’ to the statement ‘how often have you been exhausted in the past month’ while 8.1% (10/124) and 4.8% (6/124) responded ‘always’ or ‘never’, respectively (Supplementary Figure S3c). Patients with active disease were significantly more exhausted in the past month compared with patients with inactive disease (*p* = 0.007; Cohen’s *d* effect size = −0.8). Although an equal proportion of patients with active disease (27.8%, 5/18) and inactive disease (27.5%, 11/40) responded ‘mostly’ to the statement ‘how often have you been exhausted in the past month’, 0% (0/18) patients with active disease versus 10.0% (4/40) patients with inactive disease responded ‘never’ (Supplementary Figure S3c).

For more information on patients’ mental health status in the past month, as reported by patient-reported outcome measures, refer to Supplementary Figure S3.

### 3.3. Sleep

About half of the patients surveyed (53.2%, 66/124) reported that adult-onset Still’s disease had affected their sleep in the past month. There was almost an equal division of patients reporting ‘fairly good’ (45.2%, 56/124) or ‘fairly poor’ (40.3%, 50/124) sleep in the past month.

### 3.4. Work Productivity

Of 124 patients, 60.5% (75/124) reported that they were currently in paid employment. In the past year, 25.0% (31/124) patients were unable to work for <1 week–4 weeks, 17.0% (21/124) were unable to work for >1 month–≤6 months, and 9.7% (12/124) were unable to work for >6 months due to their AOSD. 41.9% (52/124) of patients had to limit or give up professional plans due to their AOSD, and 46.0% (57/124) of patients received a sickness benefit or daily sickness allowance due to being unable to work for >6 weeks due to their AOSD. Most patients (87.1%, 108/124) had never used vocational rehabilitation measures provided by their employer (retraining measures, i.e., to receive intervention from the company to enable persons with health impairments to overcome barriers to accessing, maintaining, or returning to employment).

### 3.5. Effects of COVID-19 on Patients with AOSD

#### 3.5.1. General Health

A third of patients (33.9%, 42/124) reported that because of their disease, the COVID-19 pandemic felt like a greater burden to them compared with other people, limiting their quality of life. Almost half of the patients (49.2%, 61/124) were more afraid of contracting COVID-19 than their friends/acquaintances due to having AOSD (Table 2). Almost a third of patients (32.3%, 40/124) were afraid of catching COVID-19 at their doctor’s practice or in a hospital, and almost two-thirds of patients (62.1%, 77/124) felt they were at higher risk of contracting severe COVID-19 due to their disease (Table 2). From available responses, over half of patients (55.4%, 67/121) were concerned that their treatment would weaken their immune system and put them at higher risk of contracting COVID-19; however, from these patients, most (94%, 63/67) did not discontinue treatment (Table 2). Of patients who responded that they were affected by the COVID-19 pandemic, females were more concerned about the impact of COVID-19 on their HRQoL than males (Supplementary Table S2).

#### 3.5.2. Work Productivity

Of 124 patients, 70.2% (87/124) felt that their professional activity did not change due to the COVID-19 pandemic, although 15.3% (19/124) patients felt that the COVID-19 pandemic increased their stress levels, which had a negative impact on their productivity at work. In addition, 4.8% (6/124) patients reported being afraid to lose their job because of the COVID-19 pandemic and therefore took fewer sick days, even during periods of time when they were recovering from sickness.

### 3.6. Patient Informedness

A total of 75.8% (94/124) of patients felt well informed about their disease. Most patients (88.7%, 110/124) consulted physicians or medical staff to inform themselves about their disease, whereas around two-thirds of patients (66.9%, 83/124) used Google to find information on AOSD (Supplementary Figure S4).

## 4. Discussion

The results from the PRO-AOSD survey provide insights into patients’ perspectives on the impact of AOSD on their HRQoL and work productivity, as well as the impact of the COVID-19 pandemic.

Even though there is not a universally accepted and used assessment tool for measuring AOSD activity, it is generally accepted that increased CRP levels are associated with acute and chronic inflammation [8], and PGA has been described as a valid, reliable indicator of overall disease activity and shows strong responsiveness to clinically significant changes [10,11,12]. Of the 58 patients in this study for whom CRP levels and PGA scores were available, more had inactive disease than active disease (69.0%, 40/58 versus 31.0%, 18/58), according to the classification criteria selected for this analysis.

In this survey of patients with AOSD, HRQoL was impaired in almost all domains, but particularly in those relating to physical health, and to a greater extent in patients with active disease. These findings are consistent with previous studies, in which many SF-36 domains were impaired in patients with AOSD when compared with the general population [13,17]. It has been previously reported that both patients with active and inactive AOSD experienced significantly higher levels of anxiety and depression compared with healthy controls [16]. The lack of a notable difference in mental health outcomes between active and inactive patient groups in this cohort is unsurprising, as the psychological burden of living with a chronic condition persists regardless of disease activity. This finding underscores the importance of ongoing mental health support for patients with AOSD. Overall, about a third of patients in this study did not perceive their health as excellent, or themselves to be as healthy as other people. A significant difference (*p* < 0.001) was observed in these outcomes between patients with active versus inactive disease, with a large Cohen’s *d* effect size (*d* = 1.2), which is considered clinically meaningful [25]. These results are consistent with previous studies in AOSD, in which patients perceived their current health to be lower than their ideal one and patients with active disease reported lower HRQoL than those with inactive disease [13,17].

In this cohort, a chronic disease course was reported in two-thirds (66.7%, 12/18) of patients with active disease, with half of this subgroup (50.0%) being reported as having systemic disease with joint involvement, by physicians. Joint involvement is commonly reported in AOSD with a chronic disease course, which can evolve into joint erosions and ankyloses [5,26]. Therefore, the presence of joint involvement may identify a subset of patients with AOSD at higher risk of functional disability. In this study, disease progression to a chronic disease pattern may also be a factor for the increased pain and poorer perception of health reported by patients with active disease, versus those with inactive disease.

About a third of patients felt exhausted ‘most of the time’ during the past month.

These data align with the symptoms reported for this cohort, in which 38.7% of patients reported fatigue and weakness as frequent symptoms [24]. In the existing literature, fatigue has been reported frequently in AOSD [13,27] and constitutes a substantial burden for patients [4]. Accordingly, an evaluation of fatigue may be of clinical value in the management of patients with AOSD.

Most mental health HRQoL measures did not differ significantly between patients with active versus inactive disease, suggesting that AOSD had a greater impact on physical HRQoL than mental well-being in this cohort, which is aligned with data previously reported in rheumatoid arthritis [28]. Although many patients did not describe their general health state as excellent (25.8%, 32/124), several patients (34.7%, 43/124) did not expect their health to deteriorate. It has been previously reported that patients with rheumatic disease may develop and apply coping strategies to manage their chronic disease and improve their HRQoL [29,30]; at the time of the survey, patients in this cohort had been living with AOSD for an average of 9.4 years. Therefore, it may be possible that they developed coping strategies to adapt to their chronic disease and improve their HRQoL, supporting their mental wellbeing.

Although all patients in this study were of working age, only 60.5% (75/124) were employed. The majority of patients in this cohort who were employed did not miss work over the previous week or year. These data are consistent with a previous study on AOSD, which found that the disease did not affect patients’ occupational prestige or result in substantial time lost from work [31]. However, factors such as job type, income level and workplace flexibility were not assessed. Consequently, employment retention alone may not fully reflect the broader impact of AOSD on functional status or socioeconomic well-being.

When considering the impact of COVID-19, it is important to note that the first patient completed the survey in March 2021, which is ~1 year after COVID-19 was first reported in Germany (January 2020) [32]. The pandemic was still ongoing when the survey was conducted [33]; therefore, when assessing the level of fatigue experienced by patients with AOSD in this study, factors such as COVID-19 infection should be considered. Although half of patients felt more at risk of contracting severe COVID-19 compared with their friends/acquaintances due to their AOSD, over two-thirds of patients (67.7%, 84/124) did not feel uncomfortable going to the doctor or hospital out of fear of contracting COVID-19 there. Regarding their general health, a quarter of patients (25.0%, 31/124) did not think that they became sick more easily than others; however, almost half of the patients (49.2%, 61/124) were more afraid of contracting COVID-19 than others due to having AOSD. More than half of the patients (55.4%, 67/121) in this cohort were concerned that the medication they receive for the treatment of their AOSD may weaken their immune system and put them at higher risk of contracting COVID-19; however, only 6% (4/67) of patients discontinued treatment. This is consistent with findings from a study in patients with rheumatic disease, where 43.3% patients were reportedly concerned about COVID-19, with only 5.2% discontinuing treatment [34]. A third of patients in this cohort felt that the COVID-19 pandemic burdened them more (due to their AOSD), compared with other people, and negatively impacted their HRQoL. This finding suggests that, while not all patients experienced significant psychological distress, a large proportion perceived the pandemic as exacerbating the challenges already associated with living with a chronic inflammatory disease. Evidence from studies in broader rheumatic disease populations similarly highlights the psychological burden experienced during this period [35].

In our study, most patients felt well informed about their condition, with most obtaining information from their physician/medical staff and Google as their second most favored source of information. In contrast to our study, a previous publication on rheumatic disease in Germany found that patients mainly consulted physicians and the internet (e.g., Google) for information, which is similar to this cohort; however, patients with rheumatic disease did not feel sufficiently informed about their disease [36].

Routine use of PROs may enhance the management of Still’s disease by improving communication, supporting shared decision-making, and facilitating individualized care through multidimensional insights into patients’ experiences of disease [14]. PROs provide structured information on how patients perceive their symptoms, treatment effects, and overall well-being, helping to bridge the gap between clinical assessments and patient priorities. By focusing on outcomes that matter most to patients, PROs foster engagement, adherence and self-management while supporting clinicians in delivering personalized care tailored to patients’ treatment goals [14].

The present study had some limitations. Our evaluation of patients with active versus inactive disease was limited by a relatively small sample size, as disease activity (determined by CRP and PGA scores) data were unavailable or implausible for nearly half of the patient population, thereby restricting the analysis to a smaller subset of patients. This high proportion of missing data may have introduced selection bias, and future studies with larger, more complete datasets are needed to confirm our findings. A limitation is that COVID-19 data were only available for the overall cohort and by sex, preventing treatment and age specific analyses. In addition, the ethnic and geographic diversity of this study is limited and may not be directly applicable to populations in other areas of the world. Furthermore, no specific PRO has been developed and validated in patients with AOSD, thus limiting the generalization of the results about HRQoL.

## 5. Conclusions

Patients with AOSD in this study reported impaired HRQoL, regardless of their disease activity status. Therefore, physicians should aim for a more patient-centered approach when making clinical decisions in order to improve patients’ quality of life, in addition to aiming for adequate control of disease activity, prevention of disease-related comorbidities and complications. However, additional research in larger patient populations is required to comprehensively assess HRQoL in AOSD to enhance the management of these patients.

## Figures and Tables

**Table 1:** Baseline demographics.

	Total	Active Disease	Inactive Disease	*p*-Value *
	N = 124 (100%)	*n* = 18/58 (31.0%)	*n* = 40/58 (69.0%)	
Age, years, mean (SD)	45.5 (14.7)	46.5 (14.6)	45.3 (12.3)	0.754
Gender, n (%)				0.05
Female	74 (59.7)	12 (66.7)	15 (37.5)	
Male	50 (40.3)	6 (33.3)	25 (62.5)	
Weight, kg, mean (SD)	79.0 (19.1)	79.3 (20.0)	81.1 (17.7)	0.749
Height, cm, mean (SD)	170.8 (9.9)	171.2 (7.6)	174.0 (9.1)	0.237
BMI, kg/m^2^, mean (SD)	27.0 (5.8)	26.9 (6.3)	26.7 (4.9)	0.895
Smoker, n (%)	18.0 (14.5)	2.0 (11.1)	6.0 (15.0)	1
Age at diagnosis, years, mean (SD)	32.2 (14.8) ^†^	36.0 (18.2)	38.6 (13.3)	0.604

* *p*-values were calculated using Welch test, apart from the patient’s smoking status, which were calculated using Fisher’s exact test. ^†^ Data only available for 123 patients. BMI, body mass index; n, number of patients; SD, standard deviation.

**Table 2:** Impact of the COVID-19 pandemic on patients with AOSD.

	Yes, *n* (%)	No, *n* (%)
The COVID-19 pandemic puts more strain on me as an AOSD patient than on other healthy people and further restricts my quality of life	42 (33.9)	82 (66.1)
I am more afraid than my friends/acquaintances of contracting COVID-19 because of my AOSD condition	61 (49.2)	63 (50.8)
As an AOSD patient, I feel I am at higher risk of contracting severe COVID-19	77 (62.1)	47 (37.9)
I feel uncomfortable going to the doctor or hospital and am afraid of contracting COVID-19 there	40 (32.3)	84 (67.7)
I am concerned that the medication I receive for my AOSD may weaken my immune system and put me at higher risk of contracting COVID-19 (*n* = 121)	67 (55.4)	54 (44.6)
In the past, I have stopped taking my medication to treat AOSD for this reason (*n* = 67)	4 (6.0)	63 (94.0)

AOSD, adult-onset Still’s disease.

## Data Availability

The original contributions presented in this study are included in the article/Supplementary Materials. Further inquiries can be directed to the corresponding author.

## Supplementary Materials

The following supporting information can be downloaded at: https://www.mdpi.com/article/10.3390/jcm14217848/s1, Supplementary Figure S1: Overall age at manifestation and diagnosis of AOSD (N = 124). Supplementary Figure S2: The impact of patients’ physical health status on their ability to complete everyday activities, as reported by patient-reported outcome measures. Supplementary Figure S3: Patients’ mental health status in the past month, as reported by patient-reported outcome measures. Supplementary Figure S4: Patients’ preferred sources when seeking information about their AOSD (N = 124). Supplementary Table S1: Mean scores for SF-36 domains in patients with AOSD (N = 124). Supplementary Table S2: Patients with AOSD reporting being affected by the COVID-19 pandemic.

## Abbreviations

The following abbreviations are used in this manuscript:

AID	Autoimmune disease
AOSD	Adult-onset Still’s disease
bDMARD	Biological disease modifying anti-rheumatic drugs
BMI	Body mass index
COVID-19	Coronavirus 2019
CRP	C-reactive protein
CS	Corticosteroids
csDMARD	Conventional synthetic disease modifying anti-rheumatic drugs
DMARD	Disease-modifying anti-rheumatic drug
HRQoL	Health-related quality of life
N/n	Number of patients
PGA	Physician’s Global Assessment of disease activity
PRO	Patient-reported outcomes
PRO-AOSD	Patient-reported outcomes adult-onset Still’s disease
SF-36v2	Short Form 36-item Health Survey version 2
sJIA	Systemic juvenile idiopathic arthritis
SD	Standard deviation
