# Supplementary material for: Assessment of Health-Related Quality of Life in Patients with Active Versus Inactive Adult-Onset Still’s Disease: Data from the PRO-AOSD Survey During the COVID-19 Pandemic
Source: J Clin Med. 2025 Nov 5;14(21):7848. doi: 10.3390/jcm14217848 (PMC12610029; doi:10.3390/jcm14217848)
Supplement: Supplementary file 1 [file jcm-14-07848-s001.zip › jcm-3871960-supplementary.pdf]

Assessment of health-related quality of life in patients with active versus inactive adult-onset Still's disease: data from the PRO-AOSD survey during the COVID-19 pandemic

Norbert Blank[1,2*], Ioana Andreica[3,4], Jürgen Rech[5,6,7], Zekayi Sözen[8], Eugen Feist[9,10]

[1]University Hospital Heidelberg, Division of Rheumatology, Department of Internal Medicine V, Heidelberg, Germany;

[2]University Hospital Heidelberg, Center for Rare Diseases Heidelberg (ZSEHD), Heidelberg, Germany;

[3]Ruhr-Universität Bochum, Bochum, Germany;

[4]Rheumazentrum Ruhrgebiet, Herne, Germany;

[5]Department of Internal Medicine 3 - Rheumatology and Immunology, Friedrich-Alexander University (FAU) Erlangen-Nürnberg and Universitätsklinikum Erlangen, Erlangen, Germany;

[6]Deutsches Zentrum für Immuntherapie (DZI), Friedrich-Alexander-Universität Erlangen-Nürnberg and Uniklinikum Erlangen, Erlangen, Germany;

[7]Centre for Rare Diseases Erlangen (ZSEER), Friedrich-Alexander-Universität Erlangen-Nürnberg and Uniklinikum Erlangen, Erlangen, Germany;

[8]Novartis Pharma AG, Basel, Switzerland;

[9]Helios Fachklinik Vogelsang-Gommern Klinik für Rheumatologie, Department of Rheumatology and Clinical Immunology, Gommern, Germany;

[10]Otto-von-Guericke Universität Magdeburg, Experimental Rheumatology, Magdeburg, Germany.

* Correspondence: norbert.blank@med.uni-heidelberg.de

Supplementary Figure S1 Overall age at manifestation and diagnosis of AOSD (N = 124).

a. Proportion of patients in the different age groups at manifestation of AOSD (%)

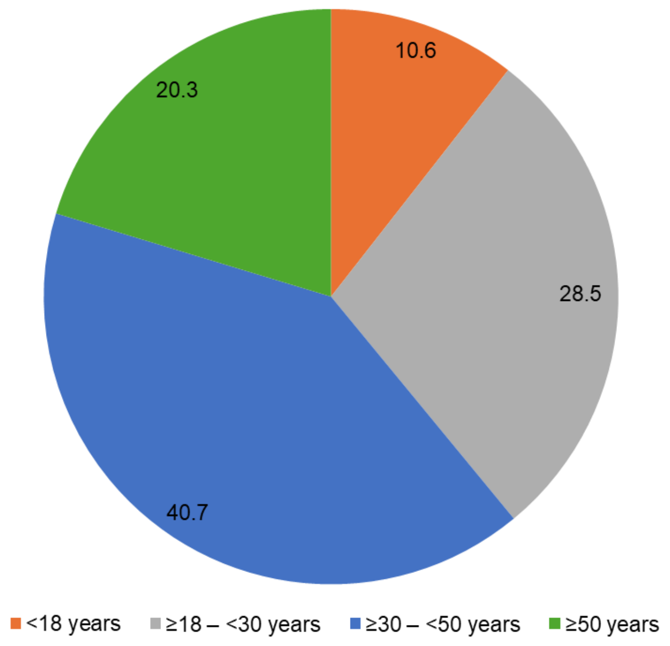

b. Proportion of patients in the different age groups at diagnosis of AOSD (%)

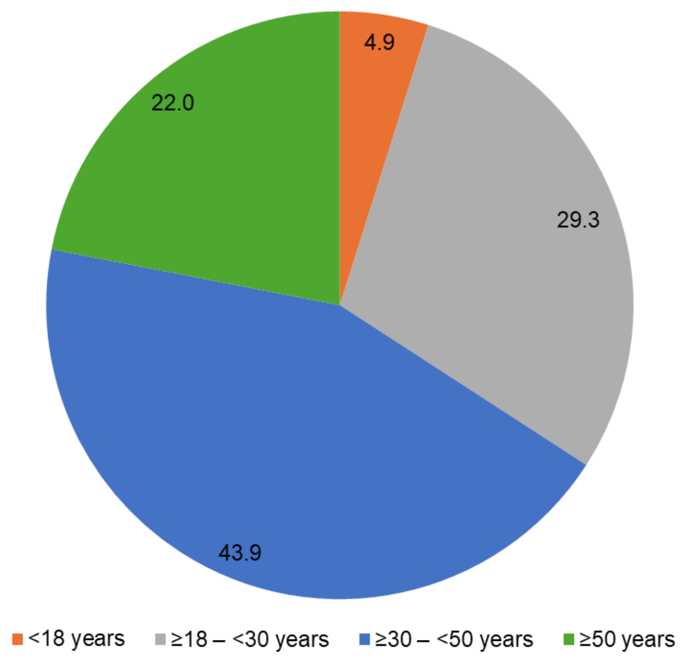

AOSD, adult-onset Still’s disease

Supplementary Figure S2 The impact of patients' physical health status on their ability to complete everyday activities, as reported by patient-reported outcome measures.

a. Does your current health condition limit your ability to bathe or dress yourself? If so, how much (Yes, severely restricted; Yes, somewhat restricted; No, not restricted at all)?

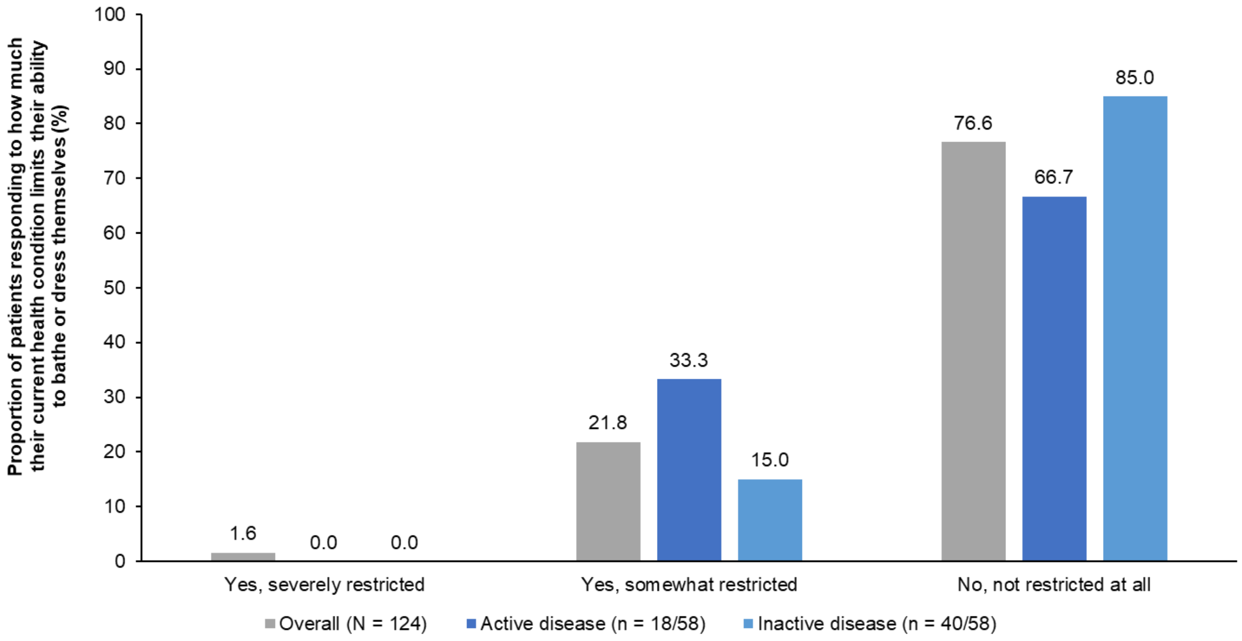

b. Does your current health condition limit your ability to lift or carry shopping bags? If so, how much (Yes, severely restricted; Yes, somewhat restricted; No, not restricted at all)?*

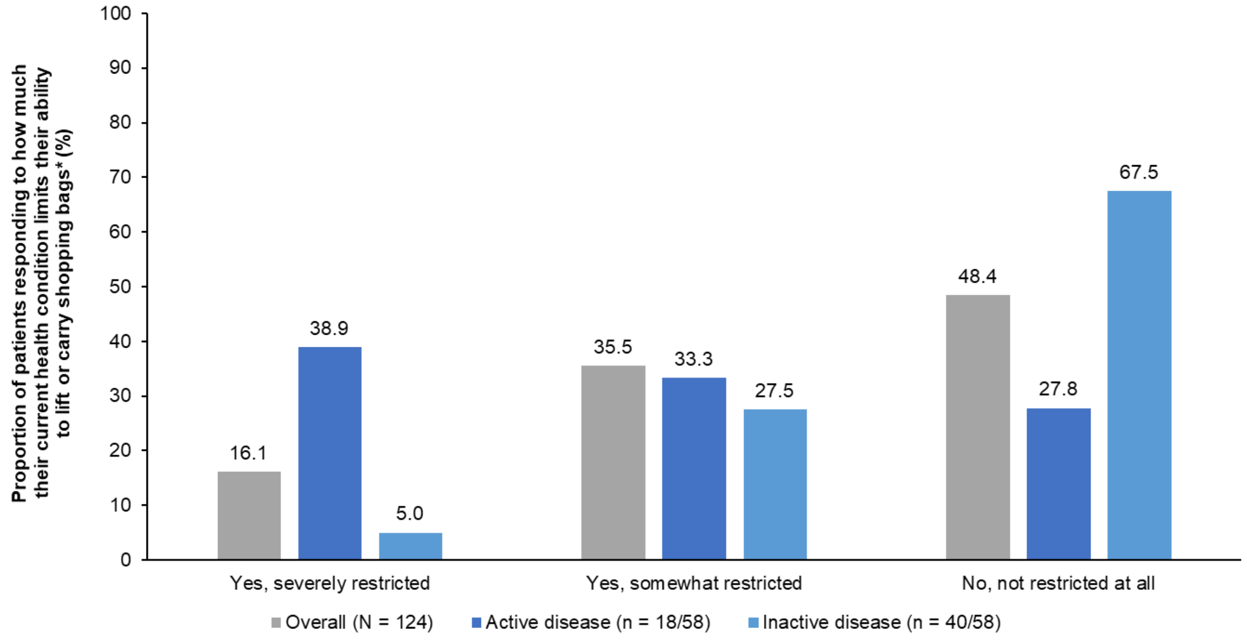

c. Does your current health condition limit your ability to bend, kneel or stoop? If so, how much (Yes, severely restricted; Yes, somewhat restricted; No, not restricted at all)?

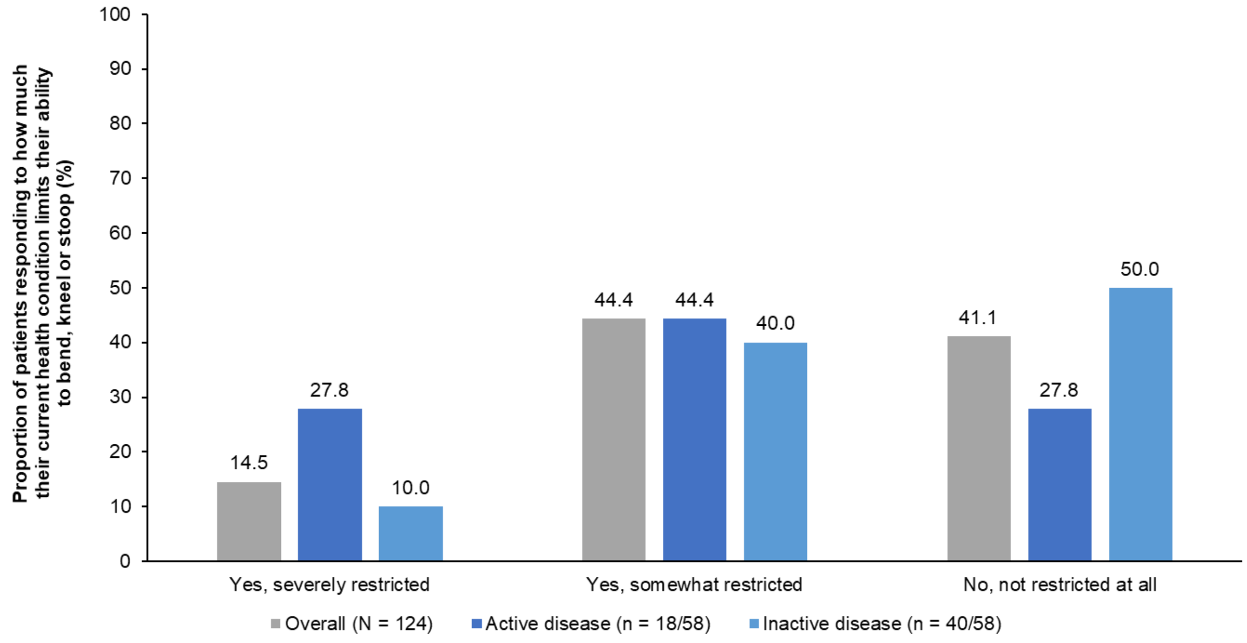

d. Does your current health condition limit your ability to perform moderately strenuous activities, such as moving a table, vacuuming, bowling, playing golf? If so, how much (Yes, severely restricted; Yes, somewhat restricted; No, not restricted at all)?†

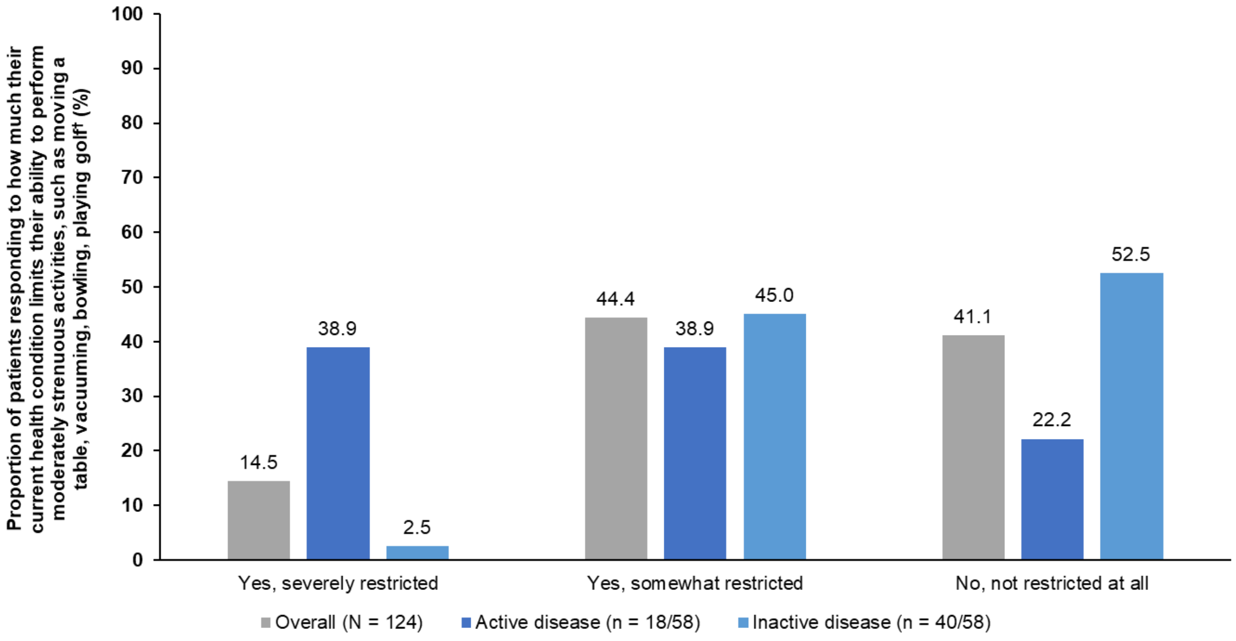

e. Does your current state of health limit your ability to perform strenuous activities, such as running quickly, lifting heavy objects, or doing strenuous exercise? If so,

how much (Yes, severely restricted; Yes, somewhat restricted; No, not restricted at all)?‡

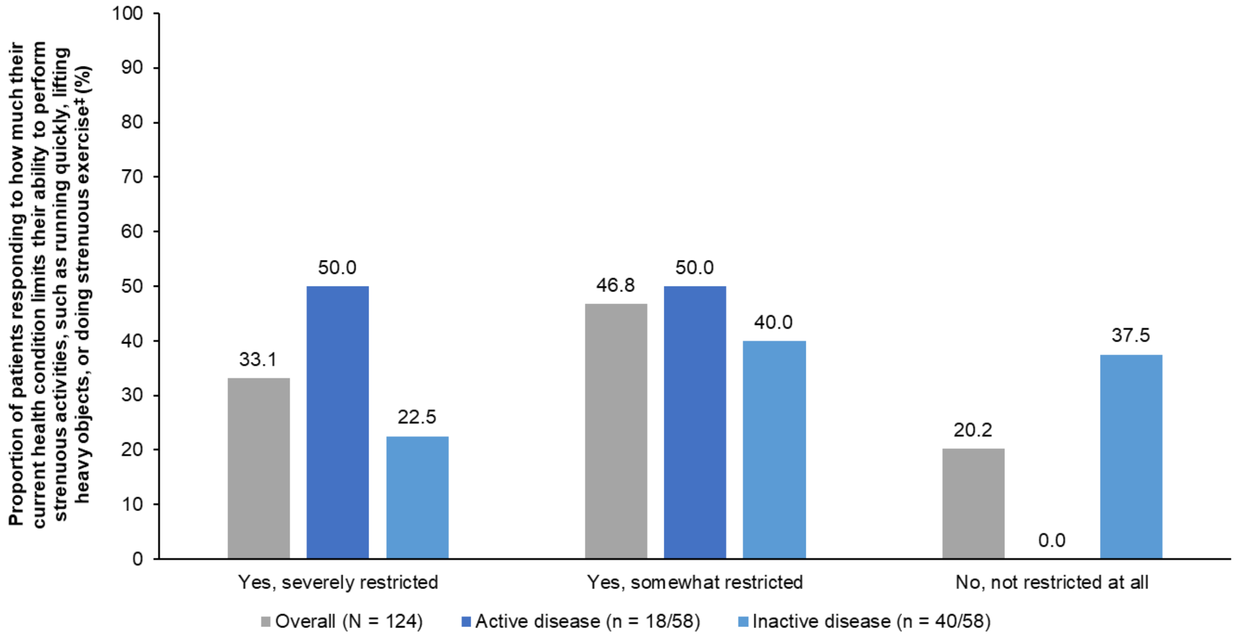

f. Does your current health condition limit your ability to climb a flight of stairs? If so, how much (Yes, severely restricted; Yes, somewhat restricted; No, not restricted at all)?†

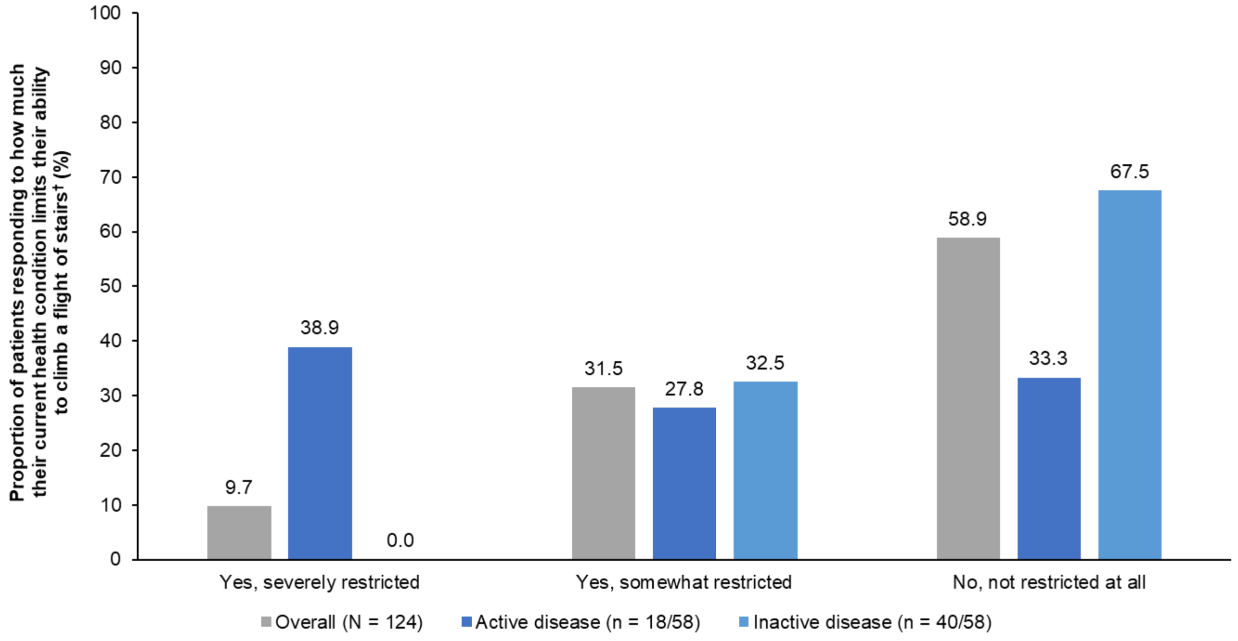

g. Does your current health condition limit your ability to climb multiple flights of stairs? If so, how much (Yes, severely restricted; Yes, somewhat restricted; No, not restricted at all)?‡

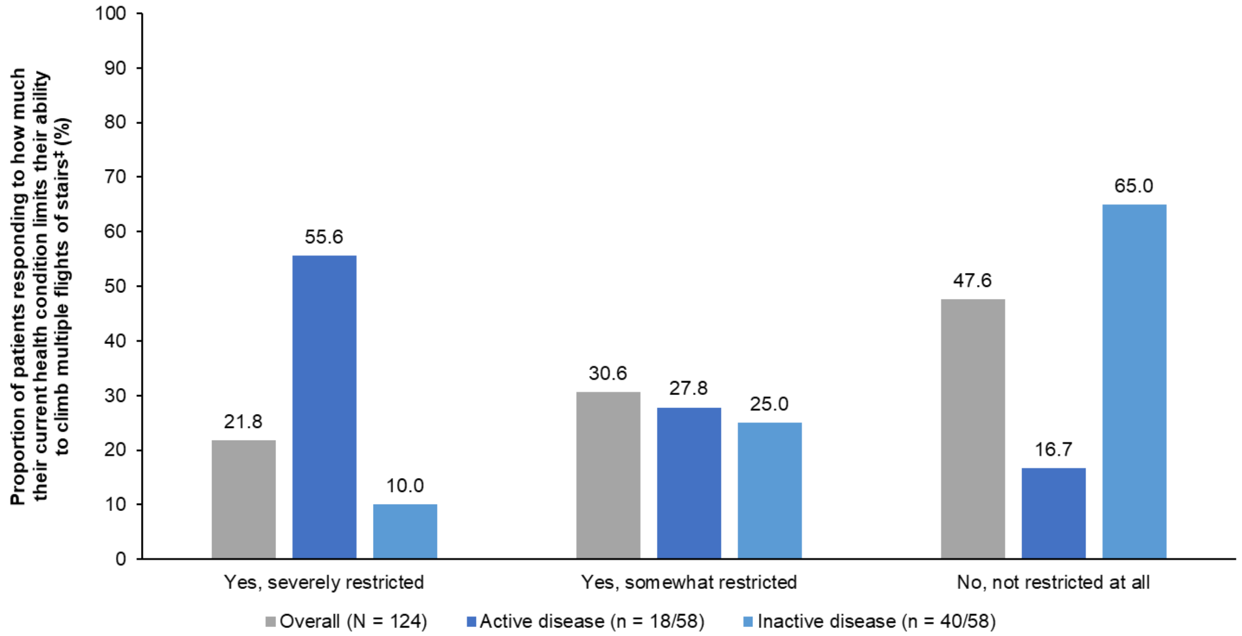

h. Does your current health condition limit your ability to walk one hundred meters? If so, how much (Yes, severely restricted; Yes, somewhat restricted; No, not restricted at all)?§

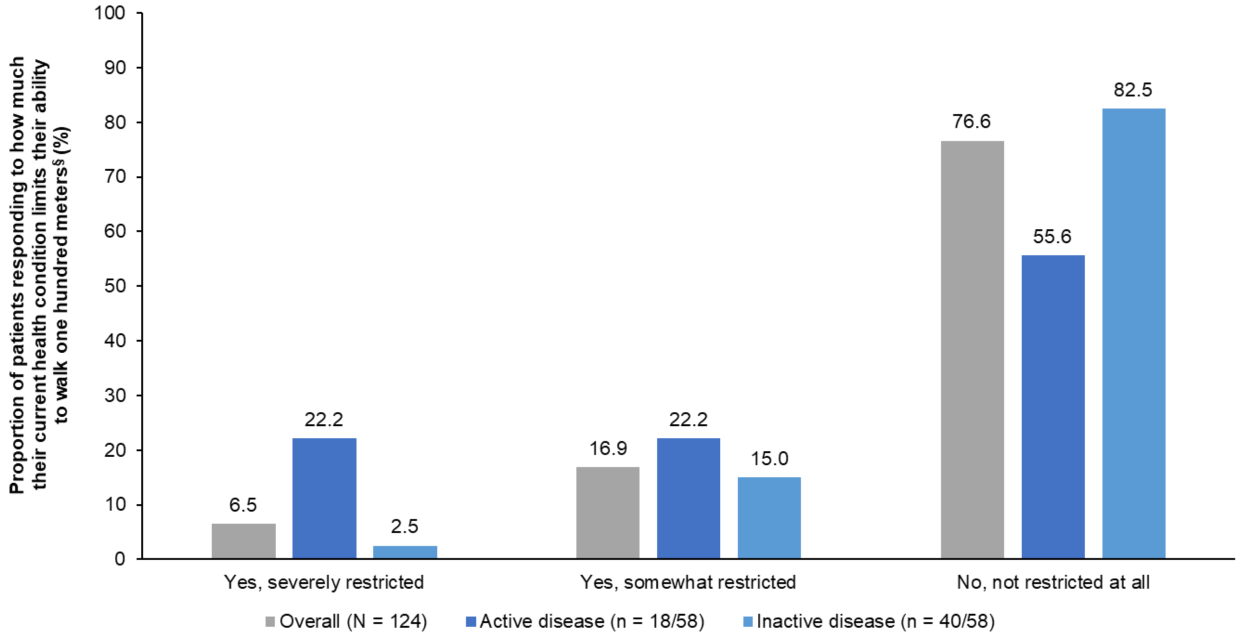

i. Does your current health condition limit your ability to walk several hundred meters? If so, how much (Yes, severely restricted; Yes, somewhat restricted; No, not restricted at all)?¶

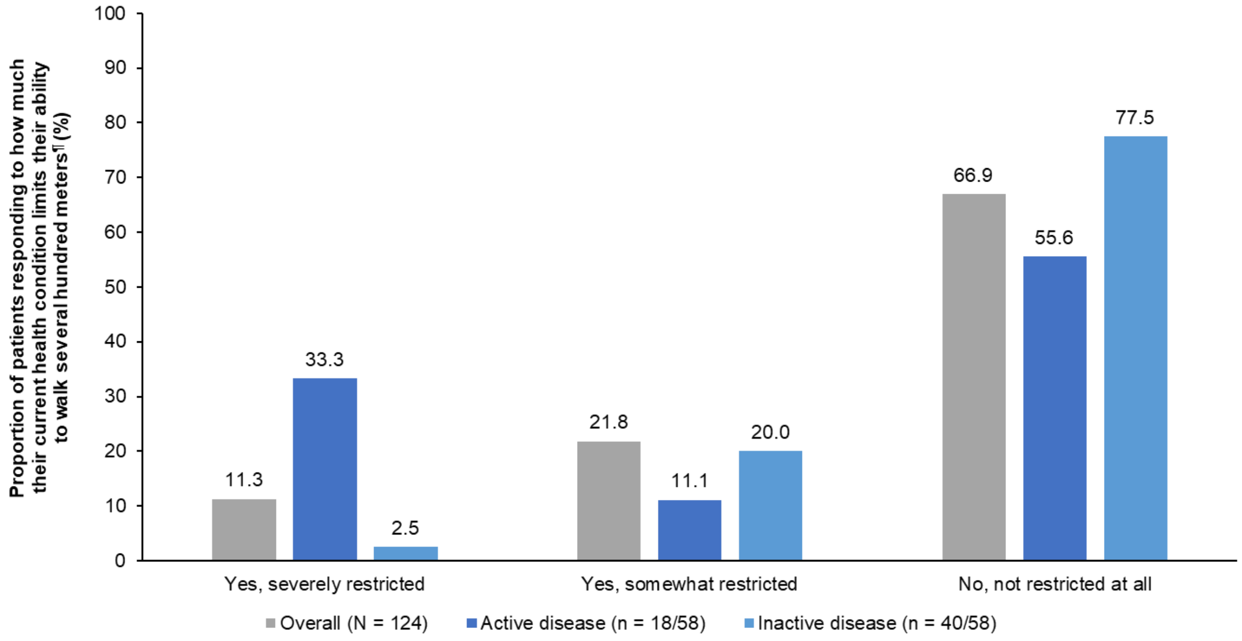

j. Does your current health condition limit your ability to walk more than one kilometer? If so, how much (Yes, severely restricted; Yes, somewhat restricted; No, not restricted at all)?#

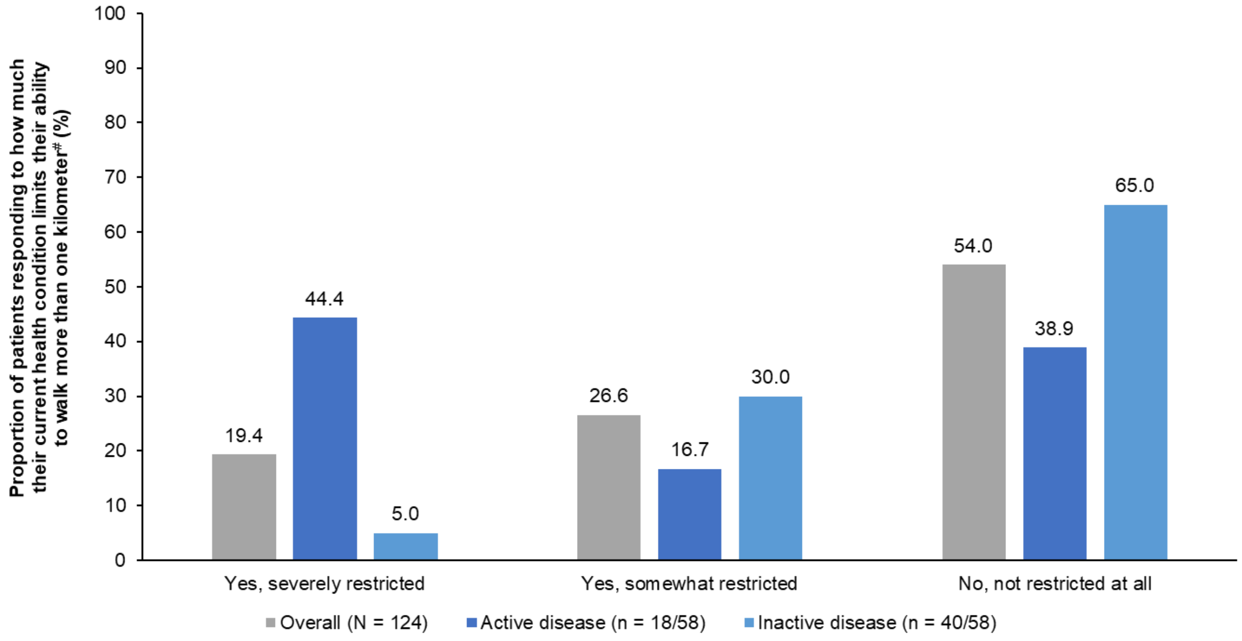

*p=0.002; [†]p=0.003; [‡]p<0.001; [§]p=0.038; [¶]p=0.036; [#]p=0.012 for active vs inactive group. Active disease was defined as CRP levels ≥10 mg/L and PGA >0, and inactive disease was defined as CRP levels <10 mg/L and PGA=0.

CRP, C-reactive protein; PGA, Physician’s Global Assessment of disease activity.

Supplementary Figure S3 Patients' mental health status in the past month, as reported by patient-reported outcome measures.

a. How often were you happy?

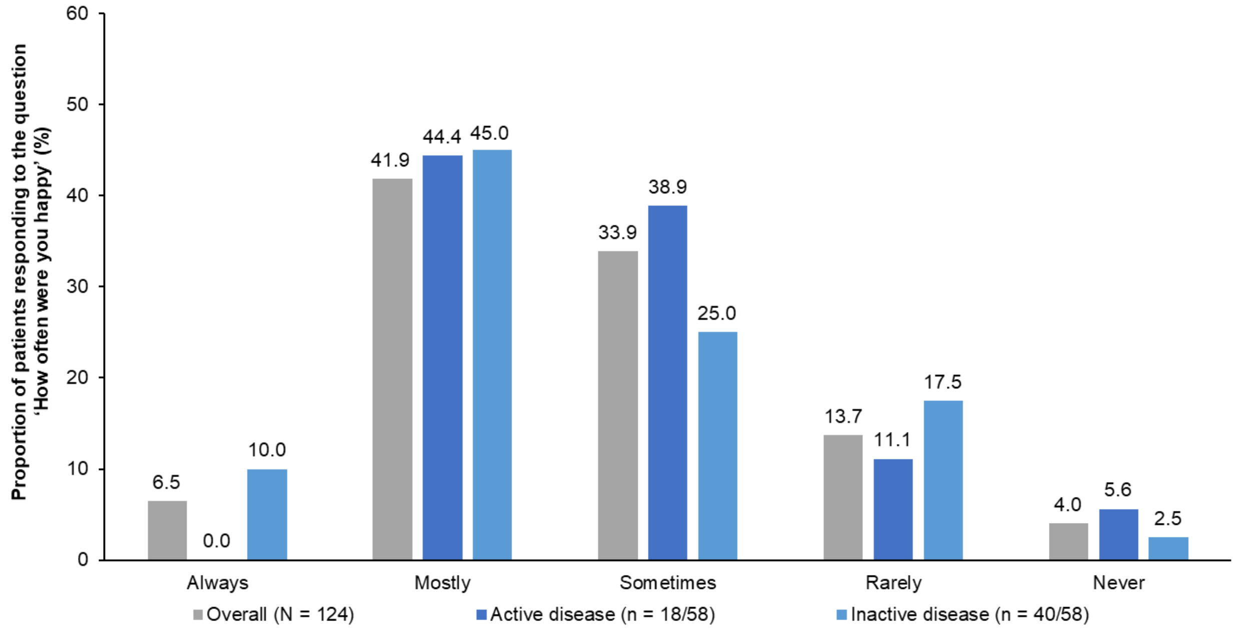

b. How often have you felt discouraged and sad?

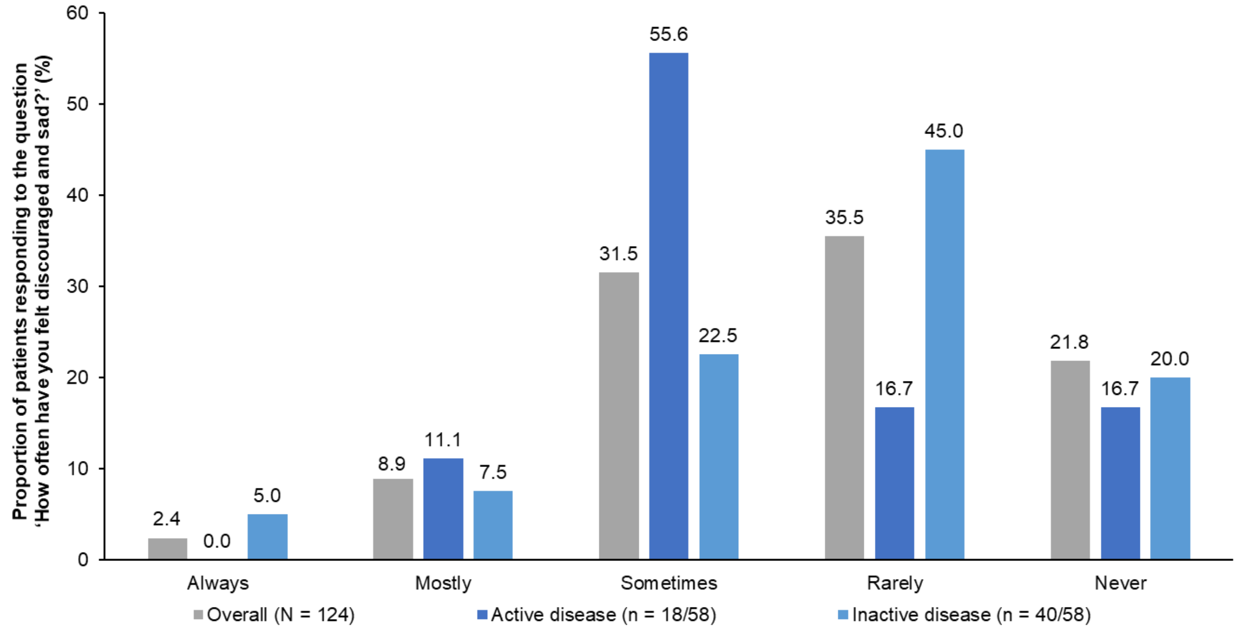

c. How often have you been exhausted?*

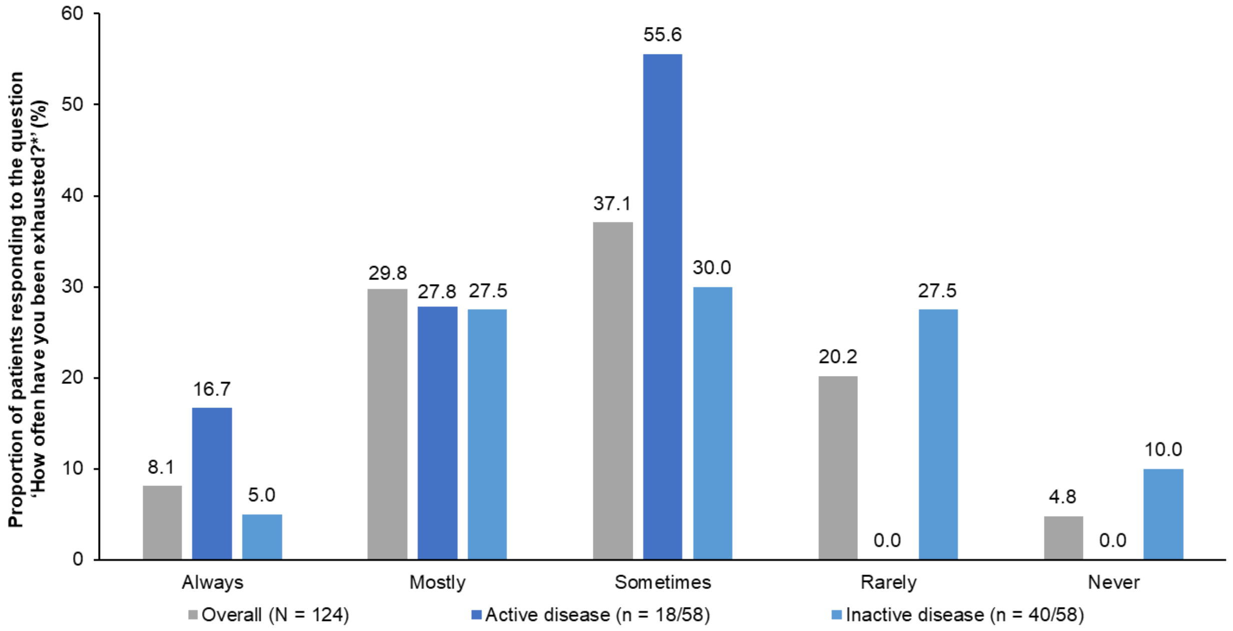

d. How often have you been calm and relaxed?

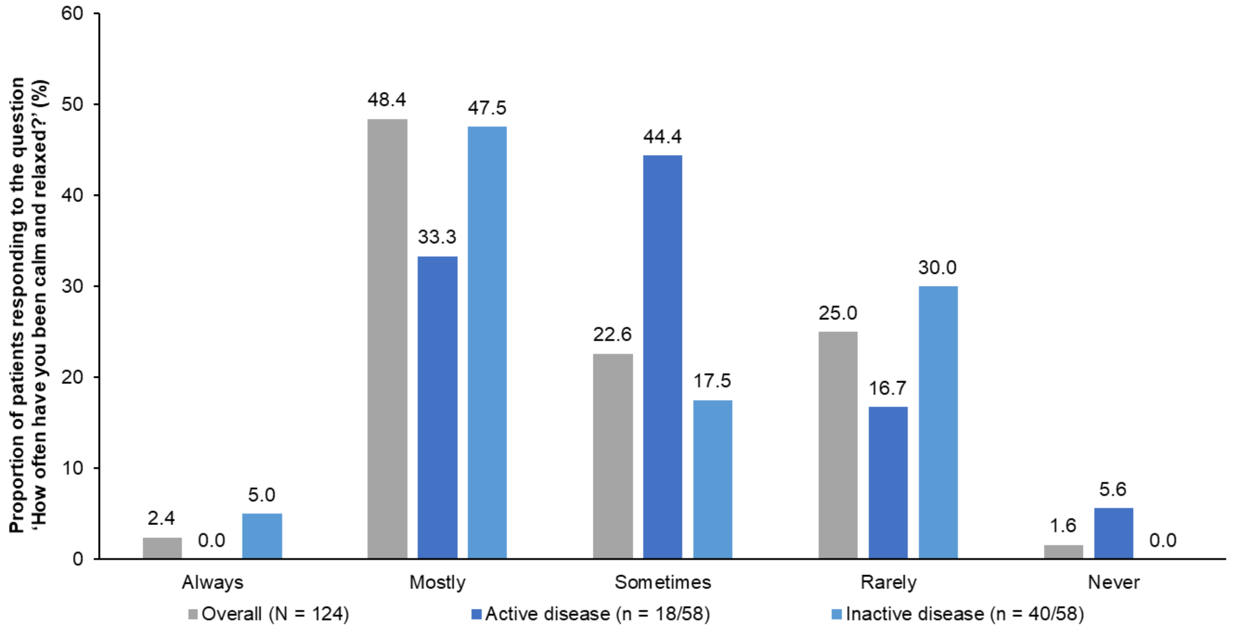

e. How often have you been full of energy?

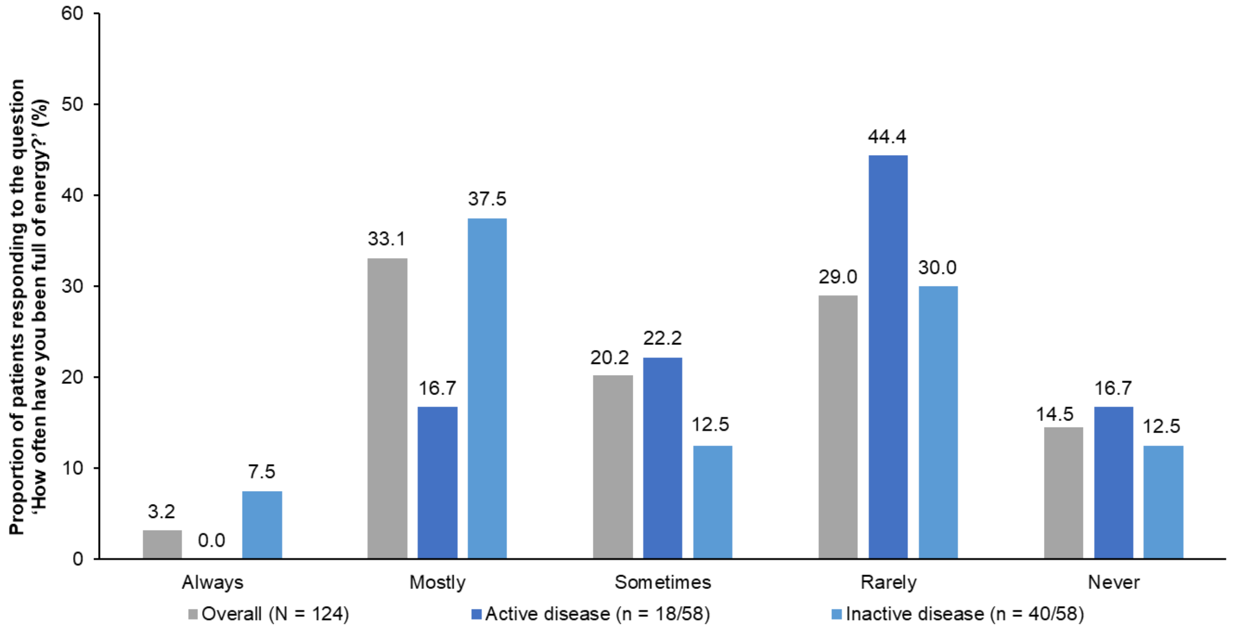

f. How often have you been full of life?

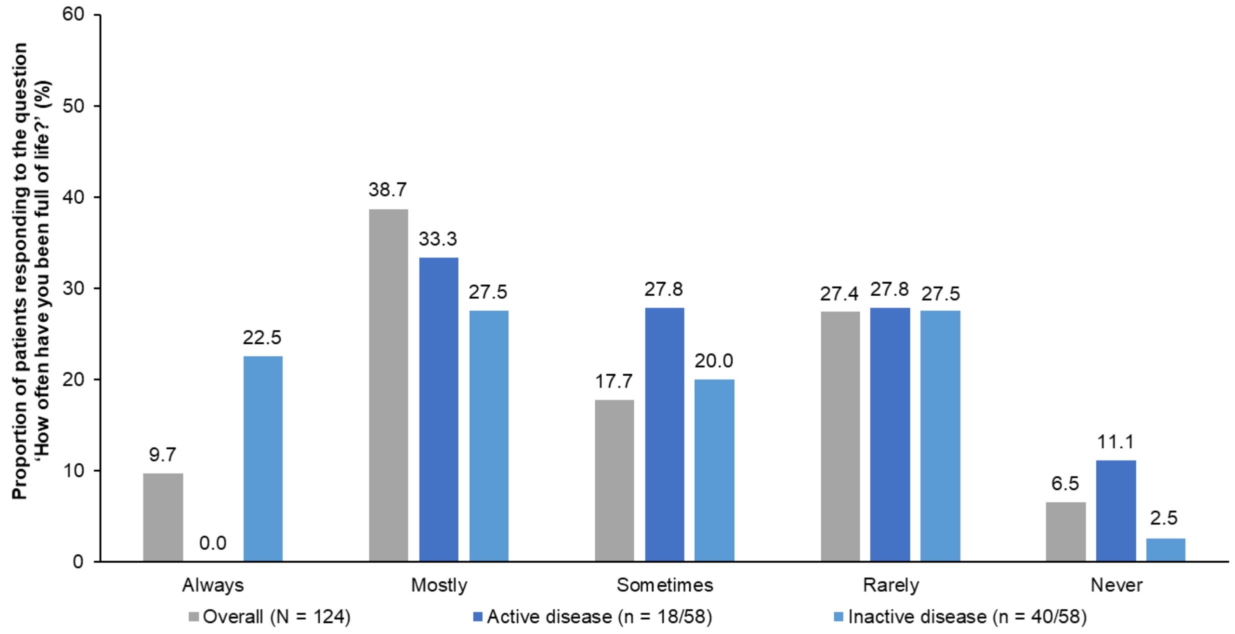

g. How often have you been tired?

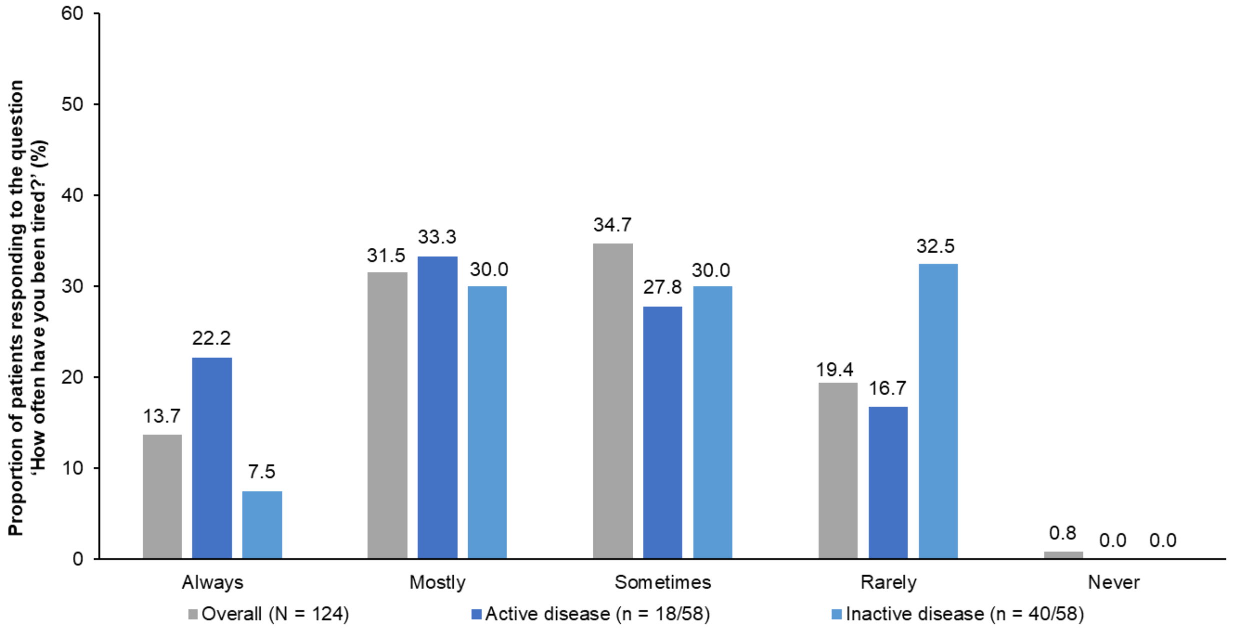

h. How often have you been very nervous?

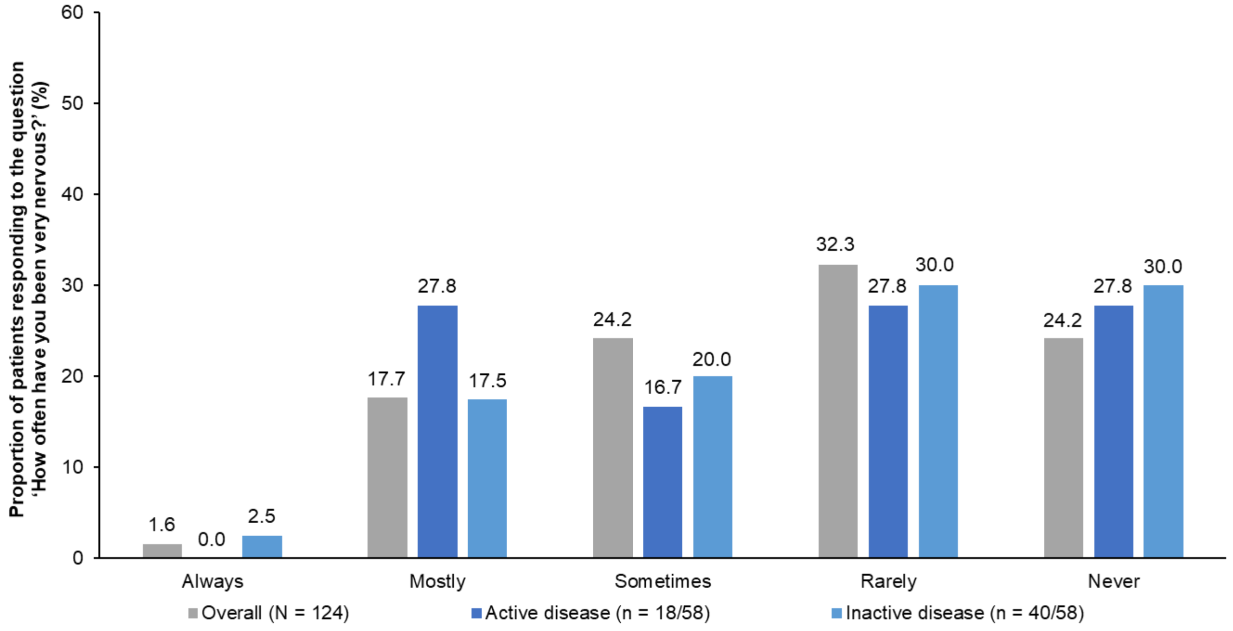

i. How many times have you been so depressed that nothing could cheer you up?

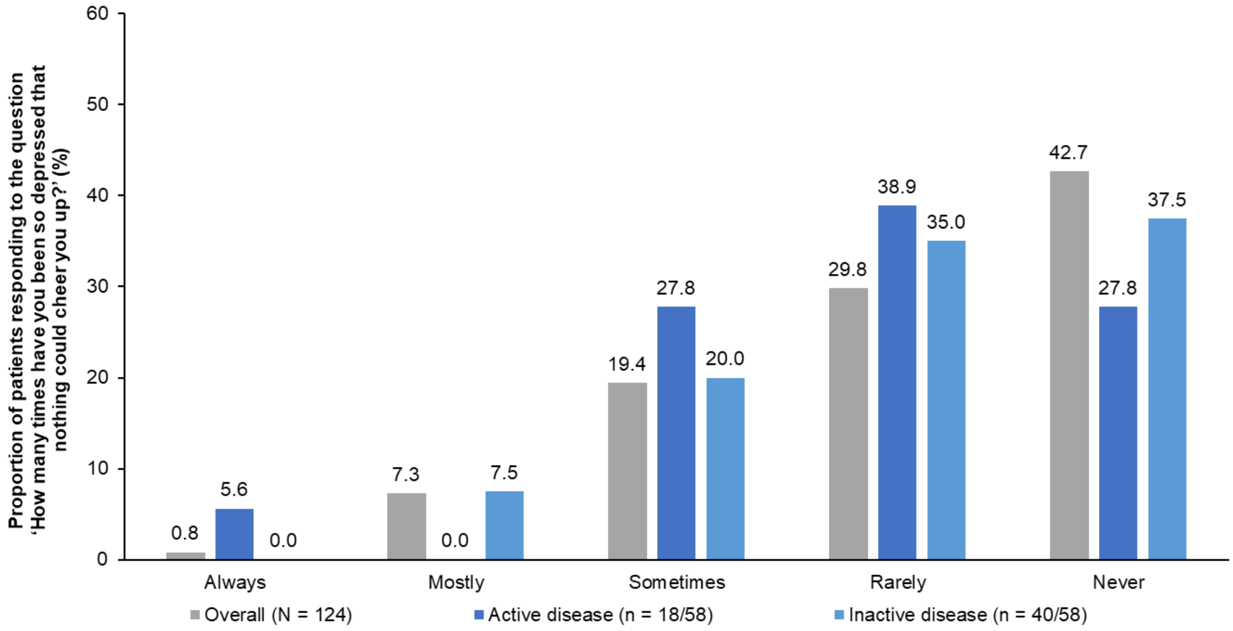

*p=0.007 for active vs inactive group. Active disease was defined as CRP levels ≥10 mg/L and PGA >0, and inactive disease was defined as CRP levels <10 mg/L and PGA=0.

CRP, C-reactive protein; PGA, Physician's Global Assessment of disease activity.

Supplementary Figure S4 Patients' preferred sources when seeking information about their AOSD (N=124).

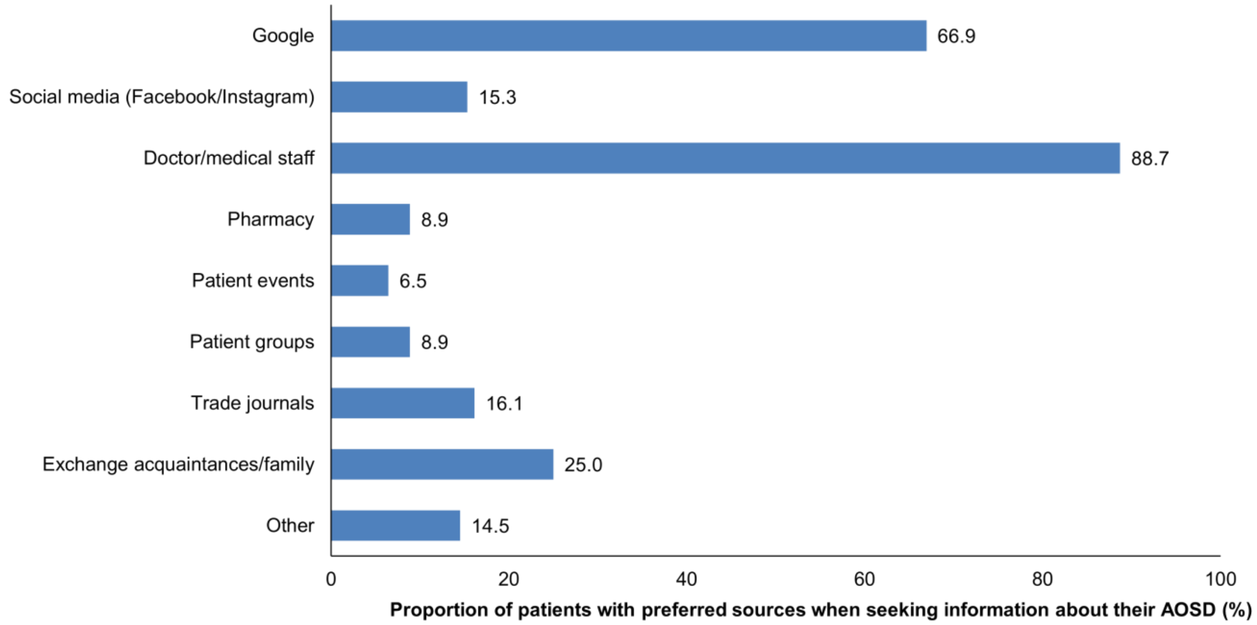

AOSD, adult-onset Still's disease; CRP, C-reactive protein; PGA, Physician's Global Assessment of disease activity.

Supplementary Table S1 Mean scores for SF-36 domains in patients with AOSD (N = 124).

	**Mean**	**Standard deviation**
**General state of health**	2.4	1.2
**Pain**	1.7	1.3
**Physical health***	2.6	1.4
**Mental health and vitality**	2.3	1.2

*Data were aggregated after separate calculations of 3-point and 5-point scale answers and normalization.

AOSD, adult-onset Still's disease

Supplementary Table S2 Patients with AOSD reporting being affected by the COVID-19 pandemic.

	Total (N=124), n (%)	Male (n=50), n (%)	Female (n=74), n (%)
**The COVID-19 pandemic puts more strain on me as an AOSD patient than on other healthy people and further restricts my quality of life**	42 (33.9)	13 (26.0)	29 (39.2)
**I am more afraid than my friends/acquaintances of contracting COVID-19 because of my AOSD condition**	61 (49.2)	24 (48.0)	37 (50.0)
**As an AOSD patient, I feel I am at higher risk of contracting severe COVID-19**	77 (62.1)	29 (58.0)	48 (64.9)
**I feel uncomfortable going to the doctor or hospital and am afraid of contracting COVID-19 there**	40 (32.3)	11 (22.0)	29 (39.2)
**I am concerned that the medication I receive for my AOSD may weaken my immune system and put me at higher risk of contracting COVID-19 (N = 121)**	67 (55.4)	22 (45.8)	45 (61.6)
**In the past, I have stopped taking my medication to treat AOSD for this reason (N = 67)**	4 (6.0)	2 (9.1)	2 (4.4)

AOSD, adult-onset Still's disease
